# Sickle-cell disease: a call to action

**DOI:** 10.1093/trstmh/trv035

**Published:** 2015-05-13

**Authors:** Frédéric B. Piel, David J. Weatherall

**Affiliations:** a Department of Zoology, University of Oxford, Oxford, OX1 3PS, UK; b MRC Molecular Haematology Unit, Weatherall Institute of Molecular Medicine, John Radcliffe Hospital, Headington, Oxford, OX3 9DS, UK

**Keywords:** Genetic disorder, Health burden, Haemoglobinopathies, Sickle-cell anaemia, World Sickle Cell Day

The inherited disorders of haemoglobin are the commonest monogenic diseases. It has been estimated that over 300 000 babies are born each year with one of these conditions, either sickle-cell disease or severe forms of thalassaemia.^1^ Normal adult haemoglobin (HbA) consists of two α- and two β-globin chains (α2β2), each with an associated heme group, which are regulated by duplicated *HBA1* genes and a single *HBB* gene, respectively. Sickle-cell haemoglobin (HbS) results from a single point mutation at position 6 in the *HBB* gene that causes the substitution of glutamic acid for valine in the β-globin chain. In conditions of reduced oxygen tension, this leads to polymerisation, a change of shape of the red blood cells, and hence to chronic anaemia, acute painful crises due to blockage of small vessels, and often to progressive damage to multiple organs, including the brain, kidneys, lungs, bones and the cardiovascular system.^2^ These complications are almost entirely restricted to those with sickle-cell anaemia, i.e., individuals who have inherited the sickle-cell mutation from both parents (SS); heterozygous carriers, who have inherited a single copy of the gene (AS), are asymptomatic, except in conditions of exceptional hypoxia.

While the clinical severity of sickle-cell disease has been clearly described, its clinical course can be extremely variable and difficult to anticipate.^3^ This is true for sickle-cell anaemia, as well as for other sickle-cell disorders such as HbSC disease or HbS β-thalassaemia, and has important implications for the development of procedures for the prevention and treatment of these disorders. Although a few genes have been identified as modifiers of the clinical course of sickle-cell disease, much work remains to be done in this important field. In contrast, the sickle-cell trait (AS) offers a very high degree of protection against infection with *Plasmodium falciparum* malaria.^4^ Recently, however, although this observation has been widely confirmed, it has been found that parasite-infected blood from sickle-cell heterozygotes is several times more infectious to the *Anopheles* vector than that of normal subjects.^5^ This suggests, of course, that the advantage of HbS heterozygotes is not only balanced by the cost of potential homozygous offspring but also by that of increased malaria transmission to the general population.

There is still some doubt about the origins of the sickle-cell mutation and where it arose for the first time. Although the hypothesis of five independent origins, four in sub-Saharan Africa (named Bantu, Benin, Cameroon and Senegal) and one in the Middle East and India (Arab-India) is almost universally accepted, both early and recent work supported the alternative hypothesis of a single origin.^6,7^ Significant differences in the level of foetal haemoglobin (HbF) between the sub-Saharan African haplotypes and the Asian might suggest that the sickle-cell mutation has arisen at least twice. While the Arab-India haplotype is usually described as associated with a milder clinical course of the disease, growing evidence indicates that its severity might have been underestimated.^8,9^

Sickle-cell disease is highly prevalent in sub-Saharan Africa, and parts of the Mediterranean region, the Middle East and the Indian subcontinent. Estimates suggest that 75% of annual conceptions with sickle-cell anaemia occur in sub-Saharan Africa and demographic projections support the fact that this share is likely to grow further in coming decades.^10^ The sickle-cell mutation was later spread to the Americas and western Europe through the trade of African slaves, and over recent decades to most countries worldwide due to increasing international movements. Universal newborn screening programmes were implemented more than a decade ago in the UK and across all states in the USA.^11^ Meanwhile, the implementation of such national programmes has not been possible in any African country, nor in India (despite recent progress, especially in the States of Gujarat, Maharashtra and Chhattisgarh), where they would have had the most impact.

Clearly, much work requires to be done towards the prevention and management of sickle-cell disease. As regards to early management, neonatal screening followed by prophylactic vaccination and antibiotics has led to a dramatic decrease in the mortality in early life in many high-income countries.^12^ There is now increasing evidence that a similar approach would be of great value in low- and middle-income countries.^13^ Regarding prenatal diagnosis and termination of pregnancy, this has been much less widely applied than in the case of thalassaemia. At least in part, this is undoubtedly due to the ethical difficulties of counselling due to uncertainties about the future clinical course of any particular patient. Clinical trials of hydroxyurea have now shown that the drug is of definite value both in adult and paediatric populations.^14^ Bone marrow transplantation has also been fairly limited for this disease, partly because of the paucity of donors and again because of the risks involved and the uncertainties in the prognosis for the disease. Nevertheless, there have been genuine improvements in the management of sickle-cell disease and it is critical that these approaches become available to the low- and middle-income countries.

Although WHO recognised sickle-cell disease as a worldwide public health issue in 2006 and a resolution on the prevention and management of birth defects, including those resulting from sickle-cell disease, was adopted a couple of years later at the 63rd World Health Assembly, a public health agenda to prevent and control this disease is still lacking in most countries of high prevalence. As recently highlighted for infectious diseases such as Ebola and HIV, there is an urgent need for better healthcare systems in low- and middle-income countries, particularly in sub-Saharan Africa. While events and celebrations organised for the World Sickle Cell Day on 19 June will hopefully contribute to raise awareness about this disease, such overall improvements in public health access and care are a prerequisite to improve the survival and quality of life of patients with sickle-cell disease in tropical regions.

## References

[1] Weatherall DJ The inherited diseases of hemoglobin are an emerging global health burden. Blood 2010;115:4331–6. doi:10.1182/blood-2010-01-251348. 20233970 10.1182/blood-2010-01-251348 PMC2881491

[2] Rees DC Williams TN Gladwin MT Sickle-cell disease. Lancet 2010;376:2018–31. doi:10.1016/s0140-6736(10)61029-x. 21131035 10.1016/S0140-6736(10)61029-X

[3] Steinberg MH Predicting clinical severity in sickle cell anaemia. Br J Haematol 2005;129:465–81. doi:10.1111/j.1365-2141.2005.05411.x. 15877729 10.1111/j.1365-2141.2005.05411.x

[4] Allison AC Protection afforded by sickle-cell trait against subtertian malareal infection. Br Med J 1954;1:290–4. 13115700 10.1136/bmj.1.4857.290 PMC2093356

[5] Williams TN Weatherall DJ World distribution, population genetics, and health burden of the hemoglobinopathies. Cold Spring Harb Perspect Med 2012;2:a011692 doi:10.1101/cshperspect.a011692. 22951448 10.1101/cshperspect.a011692 PMC3426822

[6] Flint J Harding RM Boyce AJ Clegg JB The population genetics of the haemoglobinopathies. Baillieres Clin Haematol 1993;6:215–62. 8353314 10.1016/s0950-3536(05)80071-x

[7] Bitoungui VJ Pule GD Hanchard N Beta-globin gene haplotypes among cameroonians and review of the global distribution: is there a case for a single sickle mutation origin in Africa? OMICS 2015;19:171–9. doi:10.1089/omi.2014.0134. 25748438 10.1089/omi.2014.0134 PMC4356477

[8] Alsultan A Alabdulaali MK Griffin PJ Sickle cell disease in Saudi Arabia: the phenotype in adults with the Arab-Indian haplotype is not benign. Br J Haematol 2014;164:597–604. doi:10.1111/bjh.12650. 24224700 10.1111/bjh.12650 PMC4094128

[9] Italia K Kangne H Shanmukaiah C Variable phenotypes of sickle cell disease in India with the Arab-Indian haplotype. Br J Haematol 2015;168:156–9. doi:10.1111/bjh.13083. 25135424 10.1111/bjh.13083

[10] Piel FB Hay SI Gupta S Global burden of sickle cell anaemia in children under five, 2010–2050: modelling based on demographics, excess mortality, and interventions. PLoS Med 2013;10:e1001484 doi:10.1371/journal.pmed.1001484. 23874164 10.1371/journal.pmed.1001484 PMC3712914

[11] Piel FB Patil AP Howes RE Global epidemiology of sickle haemoglobin in neonates: a contemporary geostatistical model-based map and population estimates. Lancet 2013;381:142–51. doi:10.1016/S0140-6736(12)61229-X. 23103089 10.1016/S0140-6736(12)61229-X PMC3547249

[12] Gaston MH Verter JI Woods G Prophylaxis with oral penicillin in children with sickle cell anemia. A randomized trial. N Engl J Med 1986;314:1593–9. doi:10.1056/nejm198606193142501. 10.1056/NEJM198606193142501 3086721

[13] Williams TN Uyoga S Macharia A Bacteraemia in Kenyan children with sickle-cell anaemia: a retrospective cohort and case-control study. Lancet 2009;374:1364–70. doi:10.1016/s0140-6736(09)61374-x. 19747721 10.1016/S0140-6736(09)61374-X PMC2768782

[14] Mulaku M Opiyo N Karumbi J Evidence review of hydroxyurea for the prevention of sickle cell complications in low-income countries. Arch Dis Child 2013;98:908–14. doi:10.1136/archdischild-2012-302387. 23995076 10.1136/archdischild-2012-302387 PMC3812872

